# FASN Is a Biomarker Enriched in Malignant Glioma-Derived Extracellular Vesicles

**DOI:** 10.3390/ijms21061931

**Published:** 2020-03-12

**Authors:** Franz L. Ricklefs, Cecile L. Maire, Jakob Matschke, Lasse Dührsen, Thomas Sauvigny, Mareike Holz, Katharina Kolbe, Sven Peine, Christel Herold-Mende, Bob Carter, E. Antonio Chiocca, Sean E. Lawler, Manfred Westphal, Katrin Lamszus

**Affiliations:** 1 Department of Neurosurgery, University Medical Center Hamburg-Eppendorf, 20246 Hamburg, Germany; cmaire@uke.de (C.L.M.); l.duehrsen@uke.de (L.D.); tsauvigny@gmail.com (T.S.); ma.holz@uke.de (M.H.); k.kolbe@uke.de (K.K.); westphal@uke.de (M.W.); 2 Institute of Neuropathology, University Medical Center Hamburg-Eppendorf, 20246 Hamburg, Germany; matschke@uke.de; 3 Institute of Transfusion Medicine, University Medical Center Hamburg-Eppendorf, 20246 Hamburg, Germany; s.peine@uke.de; 4 Department of Neurosurgery, Heidelberg University Hospital, 69120 Heidelberg, Germany; Christel.Herold-Mende@med.uni-heidelberg.de; 5 Department of Neurosurgery, Massachusetts General Hospital, Harvard Medical School, Boston, MA 02114, USA; BCARTER@mgh.harvard.edu; 6 Harvey Cushing Neuro-Oncology Laboratories, Department of Neurosurgery, Brigham and Women’s Hospital, Harvard Medical School, Boston, MA 02115, USA; eachiocca@bwh.harvard.edu (E.A.C.); SLAWLER@BWH.HARVARD.EDU (S.E.L.)

**Keywords:** glioblastoma, astrocytoma, extracellular vesicles, exosomes, imaging flow cytometry, biomarker

## Abstract

Extracellular vesicles (EVs) are known for their important role in cancer progression and hold considerable potential as a source for tumor biomarkers. However, purification of tumor-specific EVs from patient plasma is still an urgent unmet need due to contamination by normal host cell-derived EVs, that results in compromised analytical sensitivity. Here we identified fatty acid synthase (FASN), a key lipogenic enzyme which is highly expressed in malignant glioma cells, to be elevated in CD63- and CD81-positive EVs in glioma patient plasma samples, opening vital opportunities to sort brain tumor-specific EVs.

## 1. Introduction

Glioblastomas are the most common malignant brain tumors, and despite aggressive treatment with surgery, radiation, and chemotherapy, they are nearly universally fatal within five years [1]. Post-operatively, patients are followed by magnetic resonance imaging (MRI) to evaluate the extent of surgical resection and monitor tumor progression. However, MRI can be difficult to interpret and can fail to distinguish pseudoprogression from true progressive disease. The term pseudoprogression refers to transient post-treatment imaging changes due to inflammation and/or necrosis which cause tumors to appear larger or more contrast-enhancing on MRI scans. This phenomenon occurs in up to 30% of glioma patients after radio- and chemotherapy and at even higher rates during the course of immunotherapy [2]. Suspicious lesions require invasive biopsy or resection for diagnostic confirmation. Given the limitations of conventional imaging, there is a definite need for improved, non-invasive methods for glioblastoma monitoring, ideally by liquid biopsy.

Cancer cells, including malignant glioma cells, release extracellular vesicles (EVs) into the tumor microenvironment and bloodstream. EVs are nanoscaled bilayer lipid particles, which can either originate from multivesicular bodies that fuse with the plasma membrane (exosomes), or result from direct surface membrane budding (microvesicles) [3]. EVs carry complex biological information, consisting of soluble and transmembrane proteins, RNAs, DNA, and lipids. We and others previously demonstrated that EV cargo reflects the cell of origin and can serve as a biomarker source for malignant gliomas. Specifically, we showed that EVs derived from cultured glioblastoma (GBM) stem-like cells mirror the molecular subtype of the original tumor, being either “proneural” or “mesenchymal” [4]. In addition, EVs from the blood and cerebrospinal fluid (CSF) of glioma patients were found to contain transcripts and protein of the mutated epidermal growth factor receptor variant EGFRvIII [5,6,7] as well as mutant IDH1 transcripts and DNA [8,9]. Since CSF needs to be obtained by lumbar puncture, which is a painful, time-consuming, and hazardous procedure, analysis of blood EVs would definitely be a simpler and less invasive option for longitudinal tumor monitoring. We recently demonstrated that the total number of circulating nanoparticles is significantly increased in the blood of patients with glioblastomas and other brain tumors [10]. However, since EVs are also shed by virtually all normal host cells, bona fide tumor-derived EVs constitute only a small minority of the total EV population circulating in the blood of patients, while the majority derives from normal cells. Due to this impurity, molecular analyses are often too insensitive to detect tumor-specific alterations in bulk EV preparations. Therefore, the development of methods capable of enriching tumor-derived EVs from patient plasma is of importance.

In a previous study, we analyzed EVs derived from glioma stem-like cell (GSC) lines by mass spectrometry and discovered that fatty acid synthase (FASN) was present at high levels in EVs secreted from mesenchymal as well as proneural glioblastoma cell lines [4]. FASN catalyzes the biosynthesis of palmitate which is essential for lipid synthesis and the generation of membrane structures in rapidly dividing cancer cells. FASN is overexpressed in various types of cancer including glioblastomas and is a potential therapeutic target [11,12,13,14,15,16,17]. Moreover, FASN was shown to be contained in exosomes from adipocytes [18] and prostate cancer cells [19]. In the present study, we therefore investigated whether FASN can be detected in EVs derived from malignant glioma cells and provide evidence that it can serve as a biomarker with the potential to detect and enrich tumor-derived EVs from patient plasma, an important prerequisite for in-depth genetic, epigenetic, and transcriptional analyses that may inform clinicians on molecular aberrations in the tumor and help monitor treatment effectiveness.

## 2. Results

### 2.1. Fatty Acid Synthase (FASN) is Overexpressed in Human Glioblastomas

**Figure 1:**
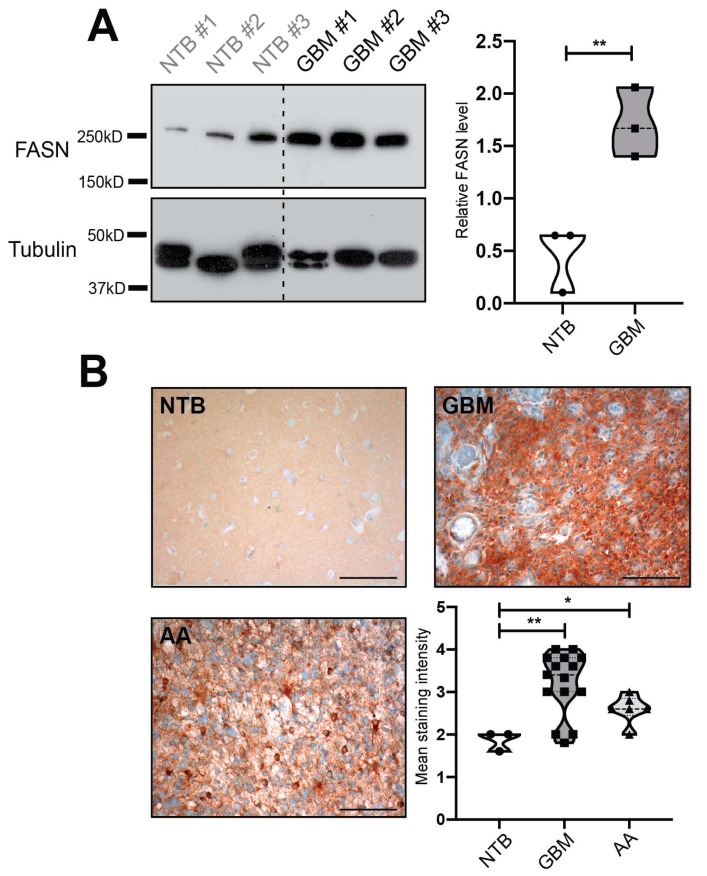
Fatty Acid Synthase (FASN) in malignant glioma tissue and non-tumorous normal brain. (**A**) Western Blot analysis of non-tumorous brain (NTB) and glioblastoma (GBM) samples. Quantification of band intensities (ratio of the optical density of FASN to tubulin) shows significantly higher expression of FASN in glioblastoma tissue than in non-tumorous brain (right panel). (**B**) FASN detection by immunohistochemistry in glioblastoma and normal brain. Samples included 15 glioblastomas, 5 anaplastic astrocytomas (AA) and 3 specimens of non-tumorous brain. Staining intensity was scored in 5 microscopic fields per sample (20× objective) as either absent (0), weak (1), moderate (2), strong (3), or very strong (4). *P* values are defined as * < 0.05 and ** < 0.01. Size bars are 100 µm.

We first analyzed the expression of FASN in malignant gliomas compared to normal non-tumorous brain tissue. Significantly elevated levels of FASN were detected in glioblastoma tissue compared to control tissue by Western blot analysis (Figure 1A). To confirm and extend this finding, we immunostained tissue samples from glioblastomas (*n* = 15), anaplastic astrocytomas (*n* = 5) and non-tumorous brain (*n* = 3) for FASN. Immunoreactivity for FASN mainly localized to the tumor cells and was usually homogeneously distributed throughout the tumor tissue (Figure 1B). In addition, prominent staining could occasionally be detected on vascular endothelial cells of intratumoral blood vessels (Figure 1B). Quantification of staining intensity confirmed a strikingly higher expression of FASN in glioblastomas than in normal brain (*P* < 0.01) and also in anaplastic astrocytomas compared to normal brain (*P* < 0.05), (Figure 1B). Further, a trend towards higher expression of FASN in glioblastomas than in anaplastic astrocytomas was observed (*P* < 0.1).

### 2.2. FASN is Expressed by Glioblastoma Cell Lines and Is Present in Secreted Extracellular Vesicles (EVs)

**Figure 2:**
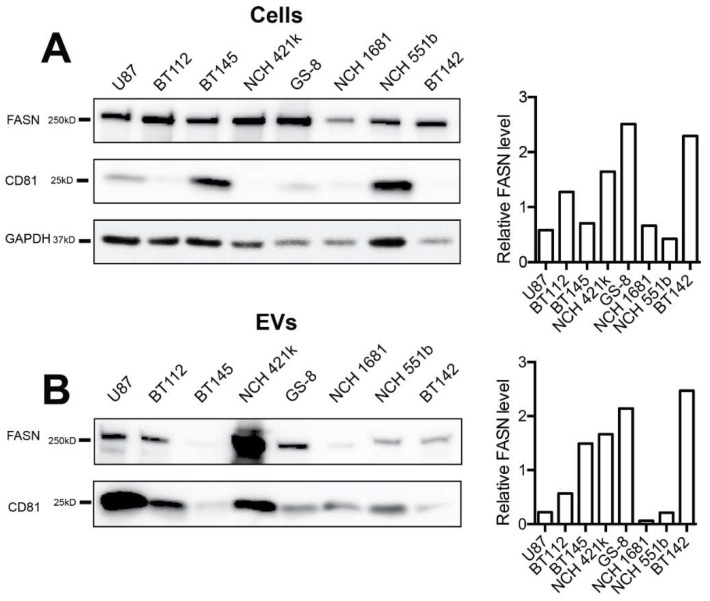
FASN expression in glioblastoma cell lines and extracellular vesicles (EVs). (**A**) Immunoblot analysis of glioma cell lines. Cells were lysed and 15 µg protein were loaded per lane. Band intensities were quantified and optical density ratios of FASN to GAPDH are shown in the right panel (**B**) Analysis of EVs secreted by glioma cell lines. EVs were isolated by ultracentrifugation and 5 µg protein were loaded per lane. CD81 was used as a reference marker for EVs, and ratios of FASN to CD81 are shown on the right.

Next, we analyzed FASN expression in a panel of glioma cell lines, including the glioma stem-like (GSC) cell lines BT112, BT145, NCH421k, and GS-8, which are IDH1-wildtype, the IDH1-mutated lines NCH1681, NCH551b, and BT142, and the serum-cultured glioblastoma cell line U87. The BT112 cell line was derived from a glioblastoma with high-level EGFR amplification and expression of the mutant receptor variant EGFRvIII, which is maintained in vitro. In all of these cell lines, we detected strong FASN expression (Figure 2A). Conditioned medium was collected from the same cell lines, and secreted EVs were isolated by ultracentrifugation. EV preparations were analyzed by nanoparticle tracking analysis (NTA), which showed that particle sizes were well within the reported range, with no significant differences between individual cell lines (data not shown). FASN was found to be contained in EVs derived from all eight cell lines, and FASN levels in EVs tended to be higher for those cell lines that displayed strong expression in their lysates (e.g., GS-8, NCH421k) than for cell lines with low expression (e.g., NCH1681, NCH551b), (Figure 2B). In addition, we analyzed the cells and EVs for CD81, a tetraspanin marker which is considered to be almost ubiquitously present on EVs from most cell types [20,21,22,23]. CD81 was detectable in lysates from six of eight cell lines and in EVs from all eight lines (Figure 2A,B). However, expression levels in cell lysates did not correlate with the amount of CD81 in EVs, a finding that is consistent with our previous observations [10] and indicates that EV packaging is driven by a specific machinery, and that the loading of tetraspanins into EVs is not random.

### 2.3. FASN is Present in EVs Circulating in the Blood of Glioblastoma Patients

**Figure 3:**
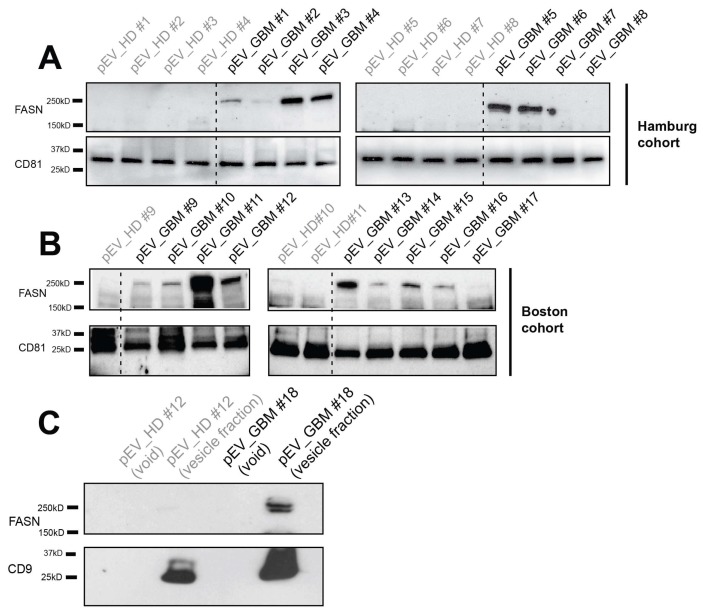
FASN is present in circulating EVs from glioblastoma patients but not from healthy controls. (**A**) EVs were purified from the plasma (pEV) of glioblastoma (GBM) patients and from age-matched healthy donors (HD) by ultracentrifugation. FASN is detected by immunoblot analysis in EVs isolated from glioblastoma patients but from healthy donors (Hamburg cohort). (**B**) Findings are confirmed in an independent cohort of patients and healthy donors collected at Brigham and Women’s Hospital, Boston. (**C**) FASN detection on circulating EVs purified by size exclusion chromatography (SEC). EVs were isolated by SEC from plasma of a healthy donor and a glioblastoma patient. FASN is only detectable in the vesicle fraction from the tumor patient (top), whereas CD9 is also present in the vesicle fraction from the healthy donor (bottom).

In order to analyze whether FASN can be detected in EVs that circulate in the bloodstream of patients with malignant gliomas, we purified EVs from patient plasma and from the plasma of healthy donors and analyzed the EV lysates by Western blotting. FASN was detectable in circulating EVs from six of eight patients of our cohort in Hamburg, whereas it could not be detected in EVs from age-matched healthy individuals (Figure 3A). This finding was confirmed by analyzing an independent cohort of samples that were collected in Boston (Figure 3B).

To exclude the possibility that FASN detection in circulating EVs might be an artefact, originating from protein aggregates that were co-precipitated by ultracentrifugation, we isolated EVs from patient plasma by size exclusion columns as an alternative technique. This analysis confirmed that FASN was exclusively detectable in EVs from glioblastoma patients but not from healthy controls and was only detectable in the vesicle fraction but not in the void (Figure 3C). In contrast, CD9 which is considered a pan-EV marker on EVs, was detectable in the vesicle fractions from both individuals (Figure 3C).

### 2.4. Detection of FASN and Tetraspanins on Single EVs by Imaging Flow Cytometry

**Figure 4:**
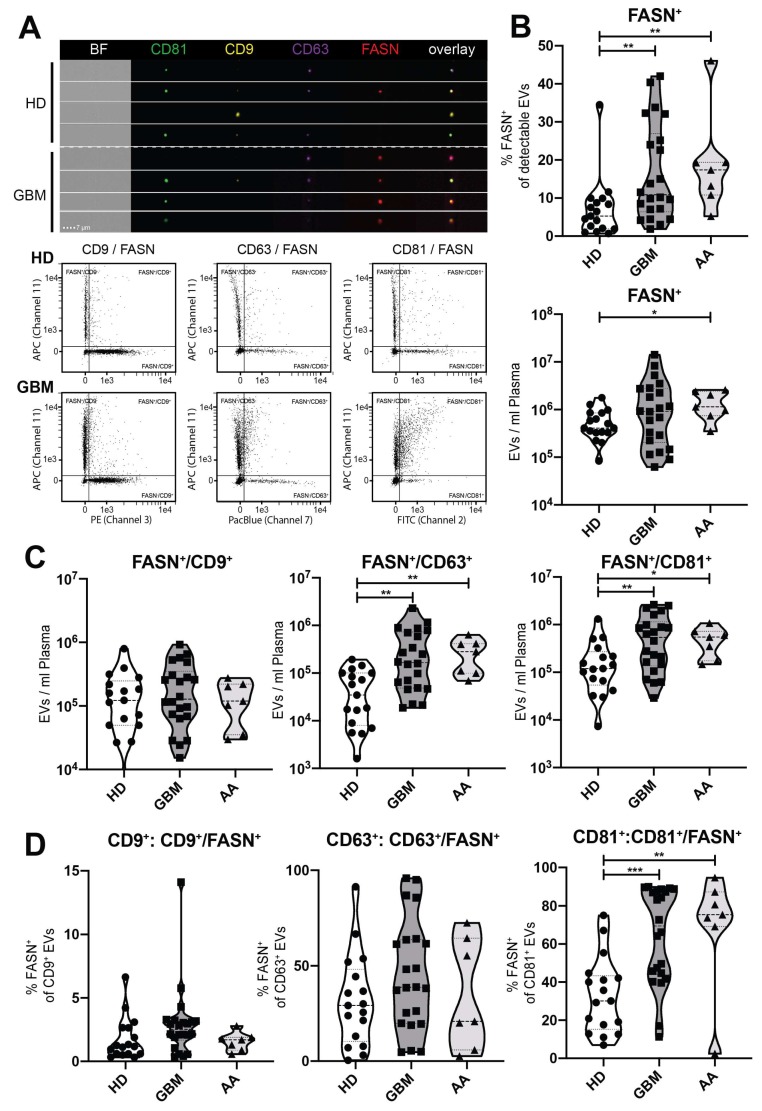
Elevated circulating FASN^+^ EV subsets in glioma patients. (**A**) Representative imaging flow cytometry (IFCM) analysis of EVs isolated by ultracentrifugation from the plasma of a healthy donor (HD) and a glioblastoma (GBM). EVs were stained with antibodies against FASN and tetraspanins as indicated. (**B**) EVs were isolated from plasma by differential ultracentrifugation and analyzed by IFCM. Left: The percentage of FASN^+^ EVs was calculated in relation to all EVs captured by either anti-CD9, -CD63 or -CD81 or combinations thereof (approximation of all total EVs). Right: Total FASN^+^ EVs per ml plasma. (**C**) Double positive FASN^+^/CD63^+^ EVs as well as FASN^+^/CD81^+^ EVs are significantly increased in the plasma of glioblastoma patients. (**D**) Percentage of FASN^+^ EVs within the EV subpopulations positive for the 3 individual tetraspanin markers. *P* values are defined as * < 0.05, ** < 0.01 and *** < 0.001. Abbreviations: GBM, glioblastoma; AA, anaplastic astrocytoma; HD, healthy donor.

We previously developed a method to characterize single tumor-derived EVs by using imaging flow cytometry (IFCM) [10]. IFCM allows the visualization of small particles (>20 nm) and simultaneous detection of the immunofluorescence labelling of multiple antibody markers. Leveraging this technique, we demonstrated in our prior study that EVs exhibiting distinctive tetraspanin profiles are secreted by different glioma and other cancer cell lines, and that CD63^+^ EVs as well as double positive CD63^+^/CD81^+^ EVs and CD9^+^/CD63^+^ EVs are enriched in glioblastoma patient plasma samples [10]. In order to assess the FASN positivity of single EVs in relation to their respective tetraspanin profiles, we performed multiplex IFCM analysis for FASN, CD9, CD63 and CD81 on EVs collected from the blood of patients with glioblastomas (*n* = 22), anaplastic astrocytomas (*n* = 7) and healthy donors (*n* = 17), (Figure 4A).

The percentage of FASN^+^ EVs in glioblastoma patients was more than twice that of healthy donors (mean 16.5% GBM vs 7.1% HD, *P* = 0.007), with a similar result for patients with anaplastic astrocytomas (mean 18.8% AA vs 7.1% HD, *P* = 0.006), (Figure 4B). In addition, the number of total FASN^+^ EVs per ml of plasma was increased in anaplastic asytrocytoma patients (mean 1.4 × 10^6^/mL AA vs 5.9 × 10^5^/mL HD, *P* = 0.04), with a similar trend in glioblastoma patients, although this did not reach significance due to a higher standard deviation (mean 2.2 × 10^6^/mL GBM vs 5.9 × 10^5^/mL HD, *P* = 0.14). Importantly, however, the total count of double positive FASN^+^/CD63^+^ EVs (mean 4.1 × 10^5^/mL GBM vs 5.8 × 10^4^/mL HD, *P* = 0.001) as well as the total count of FASN^+^/CD81^+^ EVs (mean 8.0 × 10^5^/mL GBM vs 2.3 × 10^5^/mL HD, *P* = 0.007) was significantly increased in the plasma of glioblastoma patients (Figure 4C). Similar findings were obtained for the FASN^+^/CD63^+^ EV population in anaplastic astrocytoma patients (mean 2.9 × 10^5^/mL AA vs 5.8 × 10^4^/mL HD, *P* = 0.005) as well as for the FASN^+^/CD81^+^ EV population (mean 5.2 × 10^5^/mL AA vs 2.3 × 10^5^/mL HD, *P* = 0.03), (Figure 4C).

Individual analysis of the three different tetraspanin-positive subpopulations revealed that among the subgroup of CD81^+^ EVs, a large increase in the percentage of FASN^+^ EVs occurred in glioblastoma patients compared to healthy individuals (mean 64.3% GBM vs 33.0% HD, *P* = 0.0006) and also in anaplastic astrocytoma patients (mean 69.06% AA vs 33.0% HD, *P* = 0.006) (Figure 4D). In contrast, the increase of FASN^+^/CD63^+^ EVs in glioma patients was mainly due to augmentation of the double positive EV population as a whole (Figure 4D).

## 3. Discussion

Circulating tumor-derived EVs reflect molecular alterations present in malignant gliomas and are a valuable biomarker source that can easily be obtained by liquid biopsy. However, since EVs secreted by tumor cells constitute only a minor fraction of the total EVs circulating in the bloodstream, the development of enrichment methods is crucial to facilitate sensitive detection of unknown mutations as well as comprehensive genetic, epigenetic and transcriptional profiling of glioma-derived EVs. Tetraspanin markers are commonly used to identify EVs and we previously demonstrated that the number and proportion of CD63^+^ circulating plasma EVs is significantly elevated in patients with glioblastomas and anaplastic astrocytomas, with a similar trend for CD81^+^ EVs [10]. However, tetraspanins are also present on normal host cell-derived EVs and thus of limited specificity for identifying and enriching glioma-derived EVs. We therefore investigated whether FASN can serve as a novel biomarker for glioma-derived EVs.

We detected significantly elevated levels of FASN in glioblastoma and anaplastic astrocytoma tissue compared to normal brain. This result is consistent with previous reports by other groups who demonstrated that FASN is overexpressed in gliomas vs. normal brain and correlates with malignancy grade [11,14,15,16,17]. Interestingly, FASN is also a potential drug target for the treatment of malignant gliomas. Inhibition of FASN with orlistat, cerulenin or C75 was found to reduce the viability and fatty acid synthesis of glioma cells and to induce autophagy and apoptosis in vitro [11]. In intracranial xenograft models, inhibition of FASN by using C75 or shRNA inhibited tumor growth and prolonged survival [17]. These preclinical studies highlight the functional relevance of FASN for glioma growth and metabolism.

We further found that FASN was expressed in all cultured malignant glioma cell lines that we analyzed. Importantly, FASN was detected without exception in EVs secreted by these lines. Our panel of cell lines deliberately represented a heterogeneous collection of different glioma subtypes, including both IDH1-wildtype and IDH1-mutated cells lines as well as one line that exhibits high-level EGFR amplification and expresses EGFRvIII. The universal detectability of FASN in EVs suggests that FASN is a ubiquitous biomarker in glioma-derived EVs that is not restricted to any particular subtype. In general, FASN is a tumor-associated rather than a tumor-specific antigen which is overexpressed by the majority of malignant gliomas and by the majority of individual tumor cells in these gliomas [11,14,15,16,17]. This may render FASN more widely suited biomarker for identifying glioma-derived EVs than tumor-specific antigens, such as EGFRvIII or IDH1-R132, which are only expressed in distinct subtypes and, as in the case of EGFRvIII, only by a subpopulation of tumor cells within malignant gliomas.

By immunoblot analysis, FASN could not be detected at all in EVs circulating in the blood of healthy donors. In contrast, FASN was readily detectable in the vast majority of samples (14/17) collected from glioblastoma patients. FASN is an enzyme with a high molecular weight that comprises two ~270 K multifunctional polypeptide chains with seven catalytic domains, so that it can also sediment as a protein from solutions at high ultracentrifugation force [24]. To exclude the possibility that our detection of FASN in EVs represents an ultracentrifugation artefact, we additionally used size exclusion columns as an alternative “centrifugation-independent” purification method. Thereby we confirmed that FASN is exclusively detectable in in EVs from glioblastoma patients, but not healthy donors, and is only detectable in vesicles but not in the void fraction.

We further substantiated these findings by using IFCM and quantified FASN on circulating EVs in the blood of glioma patients, relating it to tetraspanin profiles of single EVs. Notably, FASN detection on EVs does not require permeabilization and staining can readily be performed in the same buffer as tetraspanin staining, indicating that FASN antigen is exposed on the EV surface membrane, which is in line with its cellular location, i.e., the plasma membrane, cytosol, mitochondria, and other membranous organelles (https://www.genecards.org/cgi-bin/carddisp.pl?gene=FASN). Consistent with our immunoblot analysis, the percentage of FASN-positive EVs was significantly elevated in patients with glioblastomas and anaplastic astrocytomas compared to controls. However, by using IFCM as a more sensitive method, we could also detect FASN^+^ EVs in healthy donors, albeit at significantly lower amounts. We further found that total counts of double positive FASN^+^/CD63^+^ as well as FASN^+^/CD81^+^ EVs were significantly increased in the blood of glioblastoma patients, and the former was also significant in patients with anaplastic astrocytomas. These findings indicate that the joint detection of FASN together with CD63 and/or CD81 may be particularly useful for identifying tumor-derived EVs in glioma patients. Our previous study had shown that in healthy donors about 8% of the EVs circulating in the bloodstream are CD81^+^, while ~82% are CD9^+^ and only ~1% are CD63^+^. It further showed that only the total number of CD63^+^ EVs but not CD81^+^ EVs was increased in patients with glioblastomas and malignant astrocytomas. In line with these results, we now find that the rise in total FASN^+^/CD81^+^ EVs in glioma patients is mainly due to a switch from FASN negativity to FASN positivity among the CD81^+^ EV population. In contrast, the rise in FASN^+^/CD63^+^ EVs is mainly caused by an enlargement of the double positive EV population as a whole.

In conclusion, our study shows that combined marker profiling is more sensitive at detecting subtle shifts in EV subpopulations than considering only single markers and that elevated FASN^+^/CD63^+^ as well as FASN^+^/CD81^+^ EVs are characteristic of glioma patients and may help to distinguish them from healthy individuals. Future work is necessary to determine whether the increased circulating FASN^+^ EVs in glioma patients are truly derived from the tumor cells and whether tumor-specific genetic alteration can be detected in EVs enriched by FASN immunoprecipitation or other techniques. At present, it cannot be excluded that the elevated FASN^+^ EVs in glioma patients could also in part stem from other cell types in the tumor microenvironment, such as macrophages/microglia which possibly also exit the brain through a disrupted blood-brain barrier to enter the bloodstream. Another question to address in future studies is whether the increase in circulating FASN^+^ EVs is specific for gliomas or pertains also to epithelial cancers, in which FASN is frequently overexpressed and portends a poor prognosis [12,13].

## 4. Materials and Methods

### 4.1. Human Specimens

Glioma tissue, non-tumorous temporal lobe tissue from patients undergoing surgical resection for epilepsy, as well as blood samples were obtained as approved by the medical ethics committee of the Hamburg chamber of physicians (PV4904, 23rd march 2015 and PV5034, 4 August 2015) and by the Institutional Review Board at Dana-Farber Cancer Institute/Brigham and Women’s Hospital, Boston (2016P001378, 25 July 2016). Informed written consent was obtained from all patients. All experiments were performed in accordance with local guidelines and regulations. EDTA was used as anti-coagulant for all plasma samples.

### 4.2. Cell Culture

The GS-8 glioma stem-like (GSC) cell line was established from a glioblastoma using neural stem cell conditions as described previously [25]. The BT112 and BT145 glioblastoma cell lines were obtained from Dr. Keith Ligon [26]. Cell lines NCH421k and NCH551b (both glioblastomas) and NCH1681 (anaplastic astrocytoma) were established by Dr. Christel Herold-Mende [27,28]. The BT142 (oligoastrocytoma) and U87 (glioblastoma) cell lines were purchased from the ATCC.

All GSC lines (GS-8, BT112, BT145, NCH421k, NCH551b, NCH1681, BT142) were routinely cultured as neurospheres in Neurobasal Medium (NBM, Invitrogen, Carlsbad, CA, USA) supplemented with 1% Glutamine (Invitrogen), 2% B27 (Invitrogen) and 20 ng/mL each of epidermal growth factor and fibroblast growth factor–2 (PeproTech, Rocky Hill, NJ, USA), as described previously [25]. U87 cells were cultured in DMEM (Invitrogen) with 10% FBS (Invitrogen); prior to EV isolation, U87 cells were washed 3x with PBS and the medium was replaced by DMEM supplemented with Exosome-Depleted FBS (Invitrogen, #A2720801).

### 4.3. Isolation of EVs

Conditioned medium was collected from cells incubated for 48 h in fresh medium. EVs were isolated from conditioned medium or plasma by differential centrifugation as described previously [10]. Briefly, conditioned medium was centrifuged at 200× *g* for 5 min to eliminate cells, followed by filtration through 0.22 µm filters (Merck Millipore, Darmstadt, Germany). Plasma was centrifuged at 1000× *g* for 7 min. Supernatants were then further cleared by centrifugation at 10,000× *g* for 30 min. EVs were pelleted from supernatants by ultracentrifugation (TW60i, Beckman, Brea, CA, USA) at 100,000× *g* for 70 min.

Additionally, EVs were isolated using IZON qEV2 size exclusion columns, according to the manufacturer’s protocol (Izon Science, Burnside, Christchurch, New Zealand). Fractions 8–14 were collected as the vesicle fractions, while the void fraction included the first 7 fractions.

### 4.4. Imaging Flow Cytometry (IFCM) Analysis

EVs were stained in 0.22 µm filtered PBS containing 2% Exosome-depleted FBS (#A2720801, Invitrogen), supplemented with protease-inhibitor (#11836170001, Merck, Darmstadt, Germany) and phosphatase-inhibitor (#0406845001, Merck). Antibodies used to stain EVs were PE-conjugated anti-human CD9 (clone HI9a, 4 µg/mL, Biolegend, San Diego, CA, USA), PacificBlue-conjugated anti-human CD63, (clone H5C6, 40 ug/mL, Biolegend) and FITC-conjugated anti-human CD81 (clone 5A6, 40 µg/mL, Biolegend). Anti-human FASN antibody (clone C20G5, 10µg/mL, Cell Signaling, Danvers, MA, USA) was pre-labelled with the Alexa Fluor^TM^ 647 antibody labelling kit (#Z25308, Thermo Fisher, Waltham, MA, USA). EVs and antibodies were incubated in a total volume of 16 µL (with 4 µL of each antibody added to 4 µL EVs) for 45 min at 4 °C, RT and 37 °C in the dark. EVs were then washed using a 300 kDa filter (Nanosep, Pall Corporation, Port Washington, NY, USA), (4000× *g* for 7 min at 4 °C) and resuspended in washing buffer (0.22 µm-filtered PBS + 2% Exosome-depleted-FBS) for IFCM analysis. For control purposes, EVs were lysed by NP40 (0.5%) for 30 min at RT as reported previously [10].

Data were acquired on an ImageStream^X^ Mark II Imaging Flow Cytometer (Amnis/Luminex, Austin, TX, USA). Laser powers were adjusted so that the fluorescence intensity was inside the detection range or run at maximum power (details in Supplementary Materials Table S1). Fluorescent signals were collected as follows: PacificBlue was measured in channel 7 (435–505 nm filter), FITC was measured in channel 2 (480–560 nm filter), Phycoerythrin (PE) was detected in channel 3 (560–595 nm filter), and Allophycocyanin (APC) was detected in channel 11 (642–745 nm, filter). All readings were acquired at 60x magnification collected at a low flow rate and removed beads option activated. Results were analyzed as described before using IDEAS software version 6.2 [10].

### 4.5. Nanoparticle Tracking Analysis (NTA)

The concentration and size of EVs was determined by NTA, using an LM14 instrument (NanoSight, Malvern Panalytical, Malvern, UK) equipped with a 638 nm laser and a Merlin F-033B IRF camera (Adept Electronic Solutions). EV-enriched samples were diluted 1:300 in PBS prior to NTA. Quadruple one-minute movies were recorded on camera level 15 and then analyzed with detection threshold 4 in NTA 3.2 Build 16. All NTA EV size data is presented as mode values.

### 4.6. Immunoblot Analysis

Cells and EVs were lysed using RIPA buffer, containing 50 mM Tris-HCl (pH 7.5), 150 mM NaCl, aprotinin (10 mg/mL), 1 mM phenylmethylsulfonyl fluoride, leupeptin (10 mg/mL), 2 mM Na_3_VO_4_, 4 mM EDTA, 10 mM NaF, 10 mM sodium pyrophosphate, 1% NP-40, 0.1% sodium deoxycholate and 1% protease inhibitor (Merck). Total protein concentration was measured using the BCA assay. Proteins were separated using Tris-glycine gels, blotted into nitrocellulose membrane and probed with anti-FASN (#3180, 1:1000, Cell Signaling), anti-α-tubulin (sc-32293, 1:1000, Santa Cruz, Santa Cruz, CA, USA), anti-CD81 (sc-166029, 1:1000, Santa Cruz) and anti-GAPDH (#sc-20357, 1:1000, Santa Cruz) antibodies. Band intensities were quantified by densitometry using the ImageJ program version Fiji v1.52t.

### 4.7. Immunohistochemistry

Tissue specimens from patients operated in the Department of Neurosurgery, Hamburg were classified in the Institute of Neuropathology, UKE, according to the current WHO classification. Immunostaining of 5 µm thick paraffin sections was performed according to standard protocols using a Ventana Benchmark XT (Ventana, Tuscon, AZ, USA). Briefly, after antigen retrieval with citrate buffer, sections were incubated with the anti-FASN antibody (#C20G5, 1:50, Cell Signaling). Detection of secondary antibodies and counter staining was performed with an ultraview universal DAB detection kit (Ventana). Staining intensities were scored in 5 microscopic fields per sample (20x objective) as either absent (0), weak (1), moderate (2), strong (3), or very strong (4).

### 4.8. Statistical Analysis

Data are expressed as means ± SD. The unpaired two-tailed Student’s t-test was used for comparisons between groups. Each group was tested for Gaussian distribution, if this failed, the Kruskal-Wallis test was conducted. Statistical analyses were performed using Microsoft Office Excel 2011 or Graph Pad Prism 8 software. *P* < 0.05 was considered statistically significant.

## Supplementary Materials

Supplementary materials can be found at https://www.mdpi.com/1422-0067/21/6/1931/s1.
